# Supplementary material for: A disease associated mutant reveals how Ltv1 orchestrates RP assembly and rRNA folding of the small ribosomal subunit head
Source: PLoS Genet. 2023 Nov 1;19(11):e1010862. doi: 10.1371/journal.pgen.1010862 (PMC10695388; doi:10.1371/journal.pgen.1010862)
Supplement: S1 Table — (DOCX) [file pgen.1010862.s008.docx]

**Table S1: Residues with altered DMS accessibility.**

| **Residue No.** | **Average**  **WT Ltv1** | **Average**  **Ltv1_L216S** | **Δ(Ltv1_L216S vs. WT)** | **Change** |
| --- | --- | --- | --- | --- |
| 100 (A) | 0.9 | 1.6 | 0.7 | 2 bins, 1 bin |
| 103 (A) | 0.5 | 1.5 | 1.0 | 2 bins |
| 172 (C) | 0.5 | 1.8 | 1.2 | 1 bin |
| 184 (C) | 0.3 | 2.2 | 1.9 | 2 bins |
| 191 (C) | 5.1 | 2.7 | -2.4 | 2 bins, 1 bin |
| 221 (A) | 0.5 | 2.7 | 2.2 | 2 bins |
| 437 (A) | 0.5 | 1.7 | 1.2 | 2 bins |
| 990 (C) | 2.4 | 4.1 | 1.7 | 1 bin, unchanged |
| 1189 (A) | 0.4 | 1.4 | 0.9 | 2 bins |
| 1196 (A) | 0.6 | 1.4 | 0.8 | 1 bin, unchanged |
| 1197 (C) | 0.8 | 2.0 | 1.2 | 2 bins |
| 1505 (A) | 3.4 | 4.2 | 0.8 | 1 bin, unchanged |
| 1515 (A) | 1.2 | 2.2 | 1.0 | 1 bin, unchanged |
| 1591 (C) | 0.6 | 2.4 | 1.8 | 2 bins, 1 bin |
| 1753 (A) | 1.6 | 3.3 | 1.7 | 2 bins, 1 bin |
