# Supplementary material for: A disease associated mutant reveals how Ltv1 orchestrates RP assembly and rRNA folding of the small ribosomal subunit head
Source: PLoS Genet. 2023 Nov 1;19(11):e1010862. doi: 10.1371/journal.pgen.1010862 (PMC10695388; doi:10.1371/journal.pgen.1010862)
Supplement: S2 Table — (DOCX) [file pgen.1010862.s009.docx]

**Table S2: Yeast strains used in this study.**

| **Strains** | **Description** | **Genotype** | **Reference** |
| --- | --- | --- | --- |
| YKK73 | ΔLtv1 | BY4741 (MATa His3-1 Leu2-0 Met15-0 Ura3-0), Ltv1::KAN | [25] |
| YKK422 | ΔLtv1,Gal:Fap7 | BY4741, Ltv1::KAN, Gal:Fap7(NAT) | [50] |
| YKK1230 | ΔLtv1, Gal:S12 | BY4741, Ltv1::KAN; Gal:Rps12 (NAT) | This work |
| YKK1389 | ΔLtv1, snR35 | BY4741, Ltv1::NAT; snR35 (KAN) | [22] |
| YKK1576 | NOY504, ΔLtv1 | W303a (MATα, leu2-3, 112, ura3-1, trp-1, his3-11, CAN1-100); rpa12::LEU2, Ltv1::HYG | This work |
| YKK1115 | ΔLtv1, Gal:S29 | BY4741, Ltv1::HYG; Rps29B::KAN; Gal:3HA-Rps29 (NAT) | This work |
| YKK729 | ΔLtv1,Gal:S20 | BY4741, Ltv1::KAN, Gal:Rps20 (HYG) | [51] |
| YKK762 | ΔLtv1,Gal:S3 | BY4741, Ltv1::KAN, Gal:Rps3 (NAT) | [51] |
| YKK1117 | ΔLtv1,Gal:S15 | BY4741, Ltv1::KAN, Gal:Rps15 (NAT) | This work |
| YKK1625 | ΔLtv1,Gal:S31 | BY4741, Ltv1::NAT, Gal:Rps31 (HYG) | This work |
| YKK1190 | ΔLtv1,Gal:Rio2 | BY4741, Gal:Rio2(KAN), Ltv1::HYG | This work |
| YKK1142 | ΔLtv1,Gal:Enp1 | BY4741, Ltv1::KAN,Gal:Enp1(NAT) | [51] |
| YKK642 | ΔLtv1,Gal:Tsr1 | BY4741, Ltv1::KAN,Gal:Tsr1(NAT) | This work |
| YKK1184 | Gal:Rio2,Gal:Tsr1 | BY4741, Gal:Rio2(KAN), Gal:Tsr1(NAT) | [21] |
| YKK1444 | Gal:Rps31,Gal:Tsr1 | BY4741, Gal:31(HYG), Gal:Tsr1(KAN) | This work |
