# Supplementary material for: A disease associated mutant reveals how Ltv1 orchestrates RP assembly and rRNA folding of the small ribosomal subunit head
Source: PLoS Genet. 2023 Nov 1;19(11):e1010862. doi: 10.1371/journal.pgen.1010862 (PMC10695388; doi:10.1371/journal.pgen.1010862)
Supplement: S3 Table — (DOCX) [file pgen.1010862.s010.docx]

**Table S3: Plasmids used in this study.**

| **Plasmid** | **Description** | **Vector** | **Reference** | **Detailed description** |
| --- | --- | --- | --- | --- |
| PKK3350 | WT Rio2 | TEF 413 | [21] |  |
| PKK3795 | Rio2_K105E | TEF 413 | [21] | K105E; see also [52] |
| PKK30356 | Rio2_loop | TEF 413 | [21] | R129A, H133A, R136A, R139A, D140A, K143A, K144A; see also [52] |
| PKK3295 | WT Tsr1 | TEF 416 | [34] |  |
| PKK3716 | Tsr1_RK | TEF 416 | [21] | R709E,K712E |
| PKK3895 | Tsr1_Δloop | TEF 416 | [21] | Substitution of amino acids 410 to 476 with PSSGSS; see also [53] |
| PKK30183 | WT Tsr1 | TEF 415 | [21] |  |
| PKK30184 | Tsr1_RK | TEF 415 | [21] | See PKK 3716 |
| PKK30116 | S15 | TEF 416 | [21] |  |
| PKK30180 | S15_YRR | TEF 416 | [21] | Y123I,R127K,R130K |
| PKK30181 | S15_RK | TEF 416 | [21] | R137E,K142E |
| PKK3890 | WT S20 | TEF 415 | [51] |  |
| PKK3934 | S20_DE | TEF 415 | This work | D113A,E115A |
| PKK3891 | S20_EYER | TEF 415 | [51] | E80K,Y82A,E83K,R85E |
| PKK3848 | TET S20 | pCM189 | This work | Tet-off Rps20 |
| PKK3606 | WT Ltv1 | TEF 413 | [50] |  |
| PKK3607 | Ltv1_S/D | TEF 413 |  | Ltv1_S336D,S339D,S342D |
| PKK30749 | Ltv1_L216S | TEF 413 | This work | Ltv1_L216S |
| PKK30753 | Ltv1_L217S | TEF 413 | This work | Ltv1_L217S |
| PKK30643 | Ltv1 | CYC 415 | This work |  |
| PKK30762 | Ltv1_L216S | CYC 415 | This work |  |
| PKK3693 | Ltv1_ 1-394 | TEF 413 | [21] | Deletion of residues after 394. |
| PKK30750 | Ltv1_L216S_S/D | TEF 413 | This work | Ltv1_L216S and Ltv1_S336D,S339D,S342D |
| PKK30752 | Ltv1_L216S_1-394 | TEF 413 | This work | Ltv1_L216S and deletion of residues after 394. |
| PKK30266 | Ltv1_YDY | TEF 413 | This work | Ltv1_Y82A,D83R,Y84A |
| PKK31167 | S31 | GPD 416 | This work |  |
| PKK31168 | S31_Δubi | GPD 416 | This work | Deletion of the N-terminal ubiquitin |
| PKK31169 | S31_ ΔN | GPD 416 | This work | Deletion of the N-terminal extension, has ubiquitin |
| PKK3521 | WT S3 | TEF 416 | [51] |  |
| PKK3867 | S3_KK | TEF 416 | This work | S3_K7A,K10A |
| PKK3705 | S3_KR | TEF 416 | This work | S3_K75E, R76E |
| PKK4015 | TET Rps3 | pCM189 | [54] | Tet-off Rps3 |
| PKK30407 | WT S12 | TEF 415 | This work |  |
| PKK31165 | S12_R108 | TEF 415 | This work |  |
| PKK3944 | WT S29 | TEF 416 | This work |  |
| PKK3955 | TET S29 | pCM189 | This work | Tet-off Rps29 |
| PKK3541 | WT Enp1 | TEF 416 | [21] |  |
| PKK30159 | Enp1_WKK | TEF 416 | This work | Enp1_W224V,K228E,K231E |
| PKK3234 | WT 18S | pJV12 | [55] |  |
| PKK30582 | 18S_A1285A | pJV12 | [21] |  |
| PKK31082 | 18S_A1286A | pJV12 | This work |  |
| PKK30586 | 18S_A1288U | pJV12 | This work |  |
| PKK30974 | 18S_A1422U | pJV12 | This work |  |
| PKK30585 | 18S_G1288C | pJV12 | [21] |  |
| PKK30640 | 18S_C1327G | pJV12 | [21] |  |
| PKK30641 | 18S_G1288C+C1327G | pJV12 | [21] |  |
