# Supplementary material for: A disease associated mutant reveals how Ltv1 orchestrates RP assembly and rRNA folding of the small ribosomal subunit head
Source: PLoS Genet. 2023 Nov 1;19(11):e1010862. doi: 10.1371/journal.pgen.1010862 (PMC10695388; doi:10.1371/journal.pgen.1010862)
Supplement: Attachment — Submitted filename: reviewers_response.docx [file pgen.1010862.s012.docx]

We thank the reviewers for their careful and thoughtful evaluation of our work. We have now addressed all their concerns in this revised manuscript, which we believe has substantially improved its readability for a general audience. For clarity our responses are italicized.

Reviewer #1: This manuscript by Blomqvist et al. focuses on the role(s) of the ribosome assembly factor Lvt1 in 40S subunit maturation. Using yeast as a model, the authors primarily use ΔLtn1 cells and express WT or disease mutant (L216S) to determine affects on 40S maturation. The major hypothesis is that the Lvt1 L216S mutant is mispositioned on the 40S ribosome which is tested by a multitude of genetic mutants that support this hypothesis. The authors overlay the pre-40S cryo-EM and Lvt1 Alphafold structures. The position was confirmed by mutating specific putative contacts and observing synergistic or epistatic genetic interactions with yeast doubling time as a readout. The L216S mutant was positioned on the Head substructure of the pre-40S, which is highly supported by the loss (mass spec of purified 40S subunits) of primarily head subdomain RPs, but not RPs of the Platform or Body. DMS MaPSeq also demonstrated 18S rRNA changes in the Head and elsewhere, with the ladder aiding to explain downstream maturing defects. In total, the manuscript is strong, novel, and the conclusions are supported by genetic and/or biochemical evidence.

*We thank the reviewer for their positive comments on our work.*

My only stronger/major comments are:

1) It would be worth pointing out to readers whether or not expressing Lvt1 on plasmids in stains that lack endogenous Ltn1 fully or partially rescues doubling time. I don’t feel this is a big weakness as the authors nicely show the difference between WT and L216S. But it would be informative to the readers. The polysomes for the Lvt1 strain in Figure 2B (left) still has a small 40S peak suggesting there is still a 40S maturation defect.

We thank the reviewer for pointing out this question. We have included these data in the revised manuscript as Figure S1B in the supplement. Indeed the plasmid fully rescues the growth defect from Ltv1 deletion.

2) At times the nomenclature for the mutants goes back and forth of being very clear of the specific mutation (e.g., Ltv1_L216S; Rio2_K105E; S31_ ΔN) to being a bit confusing (e.g., Tsr1_RR for the R709E, K712E mutations; Ltv1_S/D for the S336D, S339D, S342D mutations. Table S3 does help with this but it is not intuitive as you read through the text. However, I can appreciate many of the mutants have already been published so changing those names would not be recommended.

*We have double checked each mutant, and whenever possible (ie not yet published), clarified the nomenclature. We have also included the actual atomic alteration in the legend to each Figure where it is used.*

3) For Figure 4F, the authors write “…demonstrate strong synthetic sick effects”. The legends notes for panel F, “Significance was tested using an unpaired t-test. ****, P<0.0001” but figure does not have markings (****) above the data to highlight this statistical test. Given that the standard deviation in these data sets must be pretty large, I am not convinced that a t test would demonstrate such a low p value or a difference that is statistically significant. Do these data accurately represent a strong effect?

*We thank the reviewer for pointing out the issues with this Figure. The yeast strain used for this experiment does not grow very well, as it makes all its rRNA from one plasmid, instead off 100-200 copies of rDNA in the yeast genome. As a result, the variation between experiments on different days is substantial. In the original manuscript, we accidentally included data from different experiments without normalizing them. When we do normalize them, then the variation is much reduced, as the effect between the wt and mutant is the same on different days (reassuringly). The revised manuscript now has normalized data, and these indeed show strong statistical significance (p<0.001). Because the normalized data does not show differences between the mutants in the background of wt Ltv1, we are now showing these data separately in Figure S3. Only data from one day of experiment are included. The second day gave the same result, but when you average the data all differences disappear. The revised section reads as follows and the revised Figure is shown below.: ”*When supplemented with plasmids encoding either wt rRNA, or a mutation of interest, their impact can then be tested. In addition, we have also supplied either wt Ltv1 or Ltv1_L216S. By themselves the rRNA mutations have moderate to no effect on growth (Figure S3). However, when combined with Ltv1_L216S, they demonstrate synthetic sick effects (Figure 4F).”

Other minor comments/suggestions/typos:

4) At times, the data are presented differently, which makes it hard to follow at times. For example, Figure 2D, some data are presented in box plots and some data are presented as (what I think are) the median and range. I think this is due to some groups having 3 data points and some having >3 data points. If other reviewers also comment on this, it may more informative and more straightforward to represented the data with the individual points but include the mean as the bar/line and error bars showing SD or SEM.

Figure 4F

*In all cases we used box-and-whisker representation, with individual data points shown. As the reviewer correctly guessed, in some cases, boxes are missing because these datasets have three data points. However, the three-datapoint representations have essentially the same information as the box-and-whisker plots, except they lack the quartile. So, the middle-line is the median, and the top and bottom-lines are the maximum and minimum, respectively. We have now clarified that in the Figure legend.*

5) On page 7, second paragraph, the authors state they tested whether Ltv1 chaperons the folding of j34-35-38 or indirectly affects its folding by preventing the premature folding of 131, or both. However, the subsequent text states the indirect effect would be inferred by an epistatic result and the direct effect would be inferred by a synergistic result. What would one expect if it is both (as the authors suggested was tested)?

*We do not think that direct effects on j34-35-38 can be well-distinguished from a model where Ltv1 acts both directly and indirectly. We have clarified this in the revised manuscript, which now reads as follows: “*The data in Figure 3D show that deletion of snR35 is synthetically sick with the Ltv1_L216S mutation, indicating that the effects on j34 misfolding arise directly from its location at j34. Nonetheless, it is difficult to rule out that additional effects might be due to premature folding of l31.”

6) On page 7, “The structure shows that Ltv1 also contacts 131 (Figure 4B), and mutation of residues Y82, D83, and Y84, which are adjacent to 131 to VAV, to make Ltv1_YDY produces a small growth defect (Figure 4C), therefore further validating the structure.” This sentence is very hard to follow in the current form.

*We thank the reviewer for pointing out the poor wording. We have now changed the text to read as follows:* “To validate this suggested interaction and test for its importance, we mutated residues Y82, D83, and Y84 to make Ltv1_YDY (Ltv1_Y82V; D83A; Y84V). This mutation produces a small growth defect (Figure 4C), therefore demonstrating the importance of this predicted interaction and further validating the structure. Moreover, this interaction between Ltv1 and l31 opened the possibility that the absence of Ltv1 leads to j34-35-38 misfolding via j34-35-38 directly, or via l31.

7) Figure 5C is first discussed after Figure 6A-G.

*Figure 5C is clearly connected to Figure 5A and B and thus was included there. However, in the narration, it makes no sense to mention it specifically beforehand, which is why this order was chosen. We hope the reviewer agrees that this helps with clarity in the presentation.*

8) Is the structure of Tsr1 also from Alphafold? Is its positioning on the pre-40S also made through molecular docking (as was done for Ltv1) or is it empirically determined?

*Tsr1’s structure has been determined by cryo-EM. We have now clarified that in the text by including the words “experimentally-determined” in front of Tsr1.*

9) In Figure 7E, the mutant is written as “Ltv1_1-394” on the x axis but written as “Ltv1_C394” in the text.

*We thank the reviewer for pointing out this discrepancy and have revised the nomenclature in the text.*

10) In Figure 7F and G, the text is on the x axis is squished.

*We thank the reviewer for pointing out this oversight and have revised the Figure.*

11) In Figure 7H, the mutant is written as “Rio2_Nloop” on the x axis but written as “Rio2_loop” in the text and legend.

*We thank the reviewer for pointing out this discrepancy and have revised the nomenclature in the Figure.*

12) In Figure 8C-F, different sized asterisks/stars are used to denote statistical significance. I believe the legends is also missing “***, P<0.001.” for panel F.

*We thank the reviewer for pointing out this discrepancy and have revised the Figure and legend.*

Reviewer #2: In this work, the authors examine the role of the ribosome assembly factor, Ltv1 using a yeast model system. Ltv1 is known to be involved in assembly of the pre-40S head domain. Previous work demonstrated that Ltv1 helps recruit r-proteins, is implicated in helping rRNA folding, and is involved in quality control during 40S maturation. Here, the authors use a disease-relevant mutation, Ltv1_L216S, which exhibits a modest growth phenotype, to characterize the functions of Ltv1. The authors begin by demonstrating that Ltv1_L216S mutation impairs 80S-like ribosome formation and inhibits Ltv1 phosphorylation, which suggests that Ltv1_L216S is mispositioned on 40S and is a useful marker for dissecting the genetic interaction networks involving Ltv1.

The authors use structural modeling and targeted mutational analyses to model Ltv1 structure in the pre-40S head domain. Strikingly, the structure predicts that Ltv1 is extended along the majority of the head domain, interacting directly with Rps3, Rps15, Rps20, Rps29, and Rps12 and h34. They carry out mass spectrometry analysis on the Ltv1_L216S ribosomes and revealed that recruitment of many RPs is impaired indicating that Ltv1 positioning is critical for RP recruitment. The authors also performed DMS-MapSeq and uncovered multiple clustered sites of altered reactivity supporting global mispositioning of Ltv1_L216S on the ribosome. Guided by the structure probing, the authors use genetic analyses to uncover that premature contact between Rps20 and Rps3 may prevent binding of other RPs, specifically Rps29. They also showed that altered DMS reactivity near the subunit interface is likely due to disruption of an interaction network between Tsr1 and Rps15. Finally, the authors show that mutations in the hinge region of Tsr1 causes the same genetic interactions with other mutations that influence Ltv1 release and that Tsr1 hinge mutations also lead to impaired recruitment of Rps31. Taken together their data suggests a mechanism in which Tsr1 movement is required to release Ltv1 and recruit Rps31.

The authors have done a lot of work and the data presented is scientifically sound. Overall, I think that the data supports their growing model for the master regulator role of Ltv1 in the late stages of 40S head assembly and will be of significant interest to the field. I have a few comments for the authors to address for clarity:

*We thank the reviewer for their careful and positive evaluation of our work.*

Major Comments:

1. For the impact of Ltv1_L216S on 80S-like ribosome formation, would the authors explain why, in Figure 2B, the amount of 18S rRNA in the 80S fractions increases relative to wt Ltv1? Is that expected?

*20S rRNA, which is the relevant species here sediments either in 40S or 80S fractions. In contrast, 18S rRNA is mostly in polysomes, which are not fully visualized in this sample. Because we loaded more sample, or more probe was used, and we are not showing the full polysomes, it appears that there is more 18S in the 80S fraction. The fraction of 18S that is translating is not decreased in wt samples.*

2. The authors compare doubling times for the mutant Ltv_S/A that abolishes phosphorylation to the Ltv1_L216S mutant, which also is not phosphorylated (Figure 2C). The difference in doubling time for the Ltv1_L216S mutant is very small compared to the difference for the Ltv_S/A mutant. Would the authors explain why the L216S mutation has only a small effect on growth when it also cannot be phosphorylated? Is there some other effect of the Ltv_S/A mutant or is there some level of phosphorylation of Ltv_L216S?

*This is a terrific question. We believe it is the latter. The assay only captures steady-state levels. The Ltv1_S/A mutant cannot be phosphorylated, as the serine is mutated. This mutant is dominant negative. In contrast, the Ltv1_L216S mutant has the small effect because its phosphorylation is impaired, and thus on average with this mutant Ltv1 is unphosphorylated.*

3. I think it is very interesting that the modeled Ltv1 structure is quite extended, spanning almost the entire head domain. I am wondering how to think about a single residue altering the network of interactions to globally effect the position of Ltv1, particularly since mutation of the neighboring residue has no growth defect (L217S). Can the authors comment on this? Have they modeled the L216S mutant protein to see if there are any predicted changes relative to wt? Also, based on their structure, would the authors comment on why L216S has an effect but L217S does not?

*We thank the reviewer for this thoughtful question. As suggested we have modeled the effect of the L216S and L217S mutations on the structure, and neither of them breaks the alpha-helix, in which they are immersed. However, the mCSM prediction tool (*[*https://biosig.lab.uq.edu.au/mcsm/results_stability_prediction/*](https://biosig.lab.uq.edu.au/mcsm/results_stability_prediction/)*) predicts a slightly larger destabilizing effect from the L216S mutation relative to the L217S mutation (-0.894 v -0.691 kcal/mol). The structure does not provide any clues, as both residues are adjacent to important elements in j34. We thus do not have a strong rationale for the differential efefcts, but note that they are consistent with the genetic results in patients.*

4. Interestingly, Rps9 is the only depleted body domain RP in the mass spec analysis of Ltv1_L216S ribosomes. The DMS data also show a cluster of altered reactivity near the Rps9 binding site. Do the authors think that this is real and significant? If so, would they comment on how Ltv1 influences Rps9 recruitment?

*The reviewer brings up an interesting point. While we cannot say for sure, we tend to discard this observation as Rps9 binds much earlier than Ltv1.*

Minor comments.

1. In the introduction – Rps5 is referred to as uS2, but I think it is uS7.

*The reviewer is absolutely correct. We thank them for pointing out this mistake.*

2. Figure 2C – I think the Ltv1-L216C label for the right panel is missing.

*I believe the reviewer is referring to Ltv1^P^. We did not add this label as there is no phosphorylated Ltv1.*

3. In Figure 4F, should there be a significance comparison as noted in the legend? The authors state in the text that when the rRNA mutations are combined with Ltv1_L216S, there is a strong synthetic sick effect, but I am not sure which ones they are referring to. For example, the mutant 18S_C1327A appears to get better when combined with the Ltv1_L216S mutation.

*We thank the reviewer for pointing out the issues with this Figure. The yeast strain used for this experiment does not grow very well, as it makes all its rRNA from one plasmid, instead off 100-200 copies of rDNA in the yeast genome. As a result, the variation between experiments on different days is substantial. In the original manuscript, we accidentally included data from different experiments without normalizing them. When we do normalize them, then the variation is much reduced, as the effect between the wt and mutant is the same (reassuringly). The revised manuscript now has normalized data, and these indeed show strong statistical significance (p<0.001). Because the normalized data does not show differences between the mutants in the background of wt Ltv1, we are now showing these data separately in Figure S3. Only data from one day of experiment are included. The second day gave the same result, but when you average the data all differences disappear. The revised section reads as follows and the revised Figure is shown in the answer to reviewer 1, question 3: ”*When supplemented with plasmids encoding either wt rRNA, or a mutation of interest, their impact can then be tested. In addition, we have also supplied either wt Ltv1 or Ltv1_L216S. By themselves the rRNA mutations have moderate to no effect on growth (Figure S3). However, when combined with Ltv1_L216S, they demonstrate synthetic sick effects (Figure 4F).”

4. Related to question 3, I am confused by the statement: “the rRNA mutations have little to no growth defect on their own (data not shown)”. Is this the data in Figure 4F as the blue bars with wt Ltv1? Can the authors also comment on the large distribution of doubling times for these mutants?

*As explained above, these strains are very tricky to get to work, and some days they will just not grow at all. The data from one day are now shown as Figure S3. The same answer but shifted to different doubling times was obtained on two additional days.*

5. I think Figure S5 and 7 have some inconsistent labels and references in the text. In Figure 7, Ltv1_1-394 is referred to as Ltv1-C394 in the text. Figure S5B is labeled as Rps15 /Rps15_1-394, should that also be Ltv1_C394? Also, in the legend for S5B, it says Ltv1_3-394 but should be Ltv1_C394. S5E also has Ltv1_1-394 in the figure, should this be Ltv1_C394?

*We thank the reviewer for pointing out these inconsistencies, which have now been corrected.*

6. Figure 9A: what do the red and green lines mean? If it is the same as in B, I would suggest getting rid of the colors on part A because it is a little confusing when comparing direct interactions versus effects of Ltv1 on assembly of these proteins.

*We thank the reviewer for this suggestion, which has now been adopted.*

Reviewer #3: Previous work in the Karbstein lab showed that the ribosome assembly factor Ltv1 facilitates correct folding of critical helix junctions in the 18S rRNA. This report takes the story one step further by investigating the effect of a mutation in Ltv1 that maps to a region of human Ltv1 associated with a rare disease. An extensive set of mutations plus DMS structure probing convincingly show that a mutation in this region of yeast Ltv1 causes Ltv1 to bind pre-40S complexes improperly, preventing Ltv1 from facilitating assembly as it normally would. The structure probing results also led to a nice connection with Tsr1 movement.

I am not wholly convinced that the genetics experiments define the order of events as unambiguously as the authors propose (see comments below). However, the abundant data presented here clearly show that the Ltv1 mutation disrupts a very delicate set of RNA and protein interactions that are essential for normal 40S assembly. Overall, this study advances the current understanding of 40S ribosome assembly in yeast.

The experiments are thorough and well done. I have some comments related to the interpretation and presentation:

*We thank the reviewer for their careful evaluation of our work.*

1. On pg. 4, human Ltv1 N168S is said to correspond directly to yeast Ltv1 L216S. However, this segment of Ltv1 is polar in mammals but hydrophobic in yeasts (Fig S1), and the sequence alignment seems tricky. Did the authors test other mutations in this region of Ltv1? It is clear from the results that the chosen mutation is consequential in yeast, but should one call it a “disease-associated mutation”?

*As indicated in the manuscript, we also tested Ltv1_L217S, mostly, because as the reviewer indicates, the sequence alignment could be ambiguous as to which of these two residues best aligns with N168S. Nonetheless, this residue does not affect yeast growth and does not show synthetic interactions with Ltv1 truncation. As indicated in the manuscript, L216 is part of an alpha-helix, and these can be both hydrophobic as well as polar.*

2. In the introduction (pg. 3) the authors state that the hierarchy of ribosomal protein binding is difficult to explain because it cannot be accounted for by direct protein-protein interactions; it must depend on conformational changes that are induced by protein binding. The concept of induced fit is well established for bacterial ribosome assembly!! The authors may want to cite papers by Noller & coworkers PMID: 2459390, 3373531 or 2658053. The order of protein addition in yeast follows the bacterial 30S assembly map, only with the insertion of eukaryotic domain RPs. Perhaps this section of the introduction could be rephrased to better convey this point.

*We thank the reviewer for pointing out the lack of clarity in this section. We have now included the suggested papers. However, the main point we were trying to make is that it is unclear why some of the proteins bind so late, not why they do not bind earlier. Delayed binding is not explained by the assembly map (which largely though not exactly follows the bacterial one), as we know from cryo-EM structures that these regions are largely folded. The revised section now reads as follows: “*The binding of ribosomal proteins (RPs) to ribosomal RNA (rRNA) is very hierarchical, as first characterized by Nomura and Nierhaus [7-9]. A potential advantage of this hierarchy is the ability to ensure that every RP is incorporated into the nascent ribosome, as the next step cannot proceed without the previous one. This is critical, as demonstrated by findings that individual RPs are often depleted in cancer cells, which is associated with a poor prognosis [10-13]. In addition, hierarchical binding can help avoid dead-end pathways, which limit the efficiency of assembly.

The small subunit head is constructed from the 18S rRNA 3’-domain and is the last of the subunit substructures to emerge. Because of its relatively late assembly, individual assembly steps can be identified using cryo-electron microscopy (cryo-EM, [14-19]), as well as biochemical investigations [20-25], rendering the head a model system to study rRNA folding [22] and RP binding *in vivo* [20-24, 26]. Its assembly is initiated co-transcriptionally with binding of Rps5 (uS7), Rps16 (uS9), Rps18 (uS13) and Rps28 [27], and then continues with binding of Rps12, Rps15 (uS19), Rps17, Rps19, Rps25 [19]. The final proteins to assemble onto the head are Rps3 (uS3), Rps10, Rps20 (uS10), Rps29 (uS14), Rps31 and Asc1 [25]. While these three groups essentially recapitulate Nomura’s description of early, middle and late binding proteins [8], a more detailed analysis of the available structural evidence indicates a more intricate assembly pathway with additional hierarchies (**Figure 1A)** that is not captured by Nomura’s original map.

Importantly, RP-induced rRNA folding [28-30] or contacts between an earlier and a later-binding RP can explain some of the hierarchy, e.g., the dependence of Rps19 binding on prior binding of Rps16 and Rps18 (**Figure 1A**). Additionally, in some cases an assembly factor blocks binding of an RP: *e.g.,* Utp15, a component of the UtpA complex, blocks premature recruitment of Rps25. However, other parts of the hierarchy are not explained in this manner: *e.g.,* it appears that the recruitment of Rps31 might be delayed, as it occurs much later than the binding if its only binding partner, Rps12, and the folding of its binding site (**Figure 1A)**. Thus, the molecular forces underpinning the hierarchical assembly of RPs remain incompletely characterized.”

When interpreting the genetics results, the authors envision a defined sequence of molecular interactions that can be over-stabilized or blocked by mutations; as I understand it, these phenotypes are used to sequence the action of Ltv1. If any mutation were to totally block a particular step, however, pre-40S complexes would pile up and the strain would be dead. In reality, many of the mutations described here have mild effects on cell growth, suggesting there is some work-around. Can the authors comment on this?

*As suggested by the reviewer, none of the mutations block assembly. Such mutations would be lethal (as for example Ltv1_S/A). We therefore were careful to indicate that these mutations “impair” assembly. In response to this comment, we have double checked this language throughout the manuscript. We believe that these mutations destabilize the conformational changes that communicate each step within the assembly cascade visualized in Figure 1B to the next. We have now clarified this point early in the Results (on the bottom of page 5) and could like to thank the reviewer for pointing out this important issue. The relevant paragraph now reads: “*Together, the data in this section demonstrate that Ltv1_L216S impairs ribosome assembly, because it cannot be phosphorylated and released, and strongly suggest that these defects arise because Ltv1 is mispositioned on pre-40S ribosomes. We also note that the small growth defects of the Ltv1_L216S mutation, as well as most of the other mutations within this work indicate that these mutations do not block the individual steps that they are associated with but rather make them less likely to occur, thereby impairing assembly.*“*

3. The alphafold model of Ltv1 was impressively useful for interpreting and designing the experiments. I appreciate the effort to validate the predicted interactions. Nevertheless, “Inspection of the structure for Ltv1 on the nascent 40S revealed…” and like phrases imply an experimental structure model, not a computational one. The text and figure legends often state that the modeled Ltv1 complex “demonstrates” or “reveals” an interaction, which of course, it cannot. It can only predict. Please rephrase such claims wherever they occur.

*We entirely agree and thank the reviewer for pointing out this misleading language, which has now been corrected throughout the manuscript.*

4. On pg. 7, the rRNA mutations are said to have no strong effect on growth but a strong synthetic effect with Ltv1_L216S. In Fig. 4, some 18S mutations in fact cause a large spread in growth rates; can the authors comment? Is it possible that the strains are picking up some second site mutations? By contrast, the synthetic effect with L216S seems modest, and generally smaller for the 18S mutants than wt 18S, which I found surprising. Have I missed something here?

*We thank the reviewer for pointing out the issues with this Figure. As described above in the responses to reviewers 1 and 2, the yeast strain used for this experiment does not grow very well, as it makes all its rRNA from one plasmid, instead off 100-200 copies of rDNA in the yeast genome. As a result, the variation between experiments on different days is substantial. In the original manuscript, we accidentally included data from different experiments without normalizing them. When we do normalize them, then the variation is much reduced, as the effect between the wt and mutant is the same (reassuringly). The revised manuscript now has normalized data, and these indeed show strong statistical significance (p<0.001). Because the normalized data does not show differences between the mutants in the background of wt Ltv1, we are now showing these data separately in Figure S3. Only data from one day of experiment are included. The second day gave the same result, but when you average the data all differences disappear. The revised section reads as follows and the revised Figure is shown in the answer to reviewer 1, question 3: ”*When supplemented with plasmids encoding either wt rRNA, or a mutation of interest, their impact can then be tested. In addition, we have also supplied either wt Ltv1 or Ltv1_L216S. By themselves the rRNA mutations have moderate to no effect on growth (**Figure S3**). However, when combined with Ltv1_L216S, they demonstrate synthetic sick effects (**Figure 4F**).”

5. Sometimes it is easier to evaluate rRNA mutations by expressing a marked copy of the 18S from the plasmid so one can avoid shutting down the chromosomal rDNA. Did the authors consider this approach?

*Because we wanted to measure growth rates, we chose to follow this approach, as only dominant mutants can be identified in the other approach. Moreover, we have recently demonstrated that partially functional mutants can be eliminated (Parker et al., BioRXiv). Together, these led us to chose the route we took.*

6. The authors conclude from the mass spec analysis that assembly of the head is generally perturbed, and I agree. In the next section, however, they interpret the relative abundance of Rps3, Asc1 and Rps20 as supporting the idea that Ltv1_L216S aberrantly stabilizes interactions between Rps3 and Rps20. I think the MS data are not precise enough to be interpreted with this level of detail because early assembly proteins (Rps9, Rps22, Rps5, Rps28, Rps16, Rps18) are depleted whereas other late assembly proteins (rpoS0) have normal stoichiometry. This goes against the idea of a specific block. This conclusion should be toned down a lot.

*We entirely agree that the mass-spec data are a reflection of both direct and indirect effects. Thus, we tried to be careful in our choice of language, and merely state that the observation that Rps3, and Rps20 are the only proteins* not *depleted is “consistent” with their premature stabilization. Like the reviewer we do not think that the data “support” or “confirm” the conclusion that the part of Ltv1 that is predicted to separate Rps3 from Rps20 (based on the structure) is repositioned. This conclusion is based on the DMS MapSeq data. The suggestion from the alpha-fold structure that Ltv1 blocks the contact between Rps3 and Rps20 is supported by previous genetic data, which suggested exactly this model (Collins et al., 2017).*

7. The data in Fig. 6 showing that addition of Rps29 (uS14) is impaired by Ltv1_L216S is very convincing. However, I had trouble following the logic of why Rps29 depletion should have a smaller effect in the Ltv1_L216S background than the wt background. If Ltv1_L216S tends to mask the binding site for Rps29, then low levels of Rps29 should make this even worse (synthetic), because the probability of Rps29 recruitment is reduced even further. Conversely, by this logic, Rps29 over-expression should rescue Ltv1_L216S. Since they observe the opposite, I wonder if the model is right. Perhaps I missed something?

*We thank the reviewer for pointing out the lack of clarity in the manuscript. We believe that the genetic data support the model, as they suggest that indeed Rps29 binding is no longer dependent on Rps29 concentration, consistent with a conformational step, which is concentration-independent. In the mutant Rps29 cannot bind as its site is blocked by Rps20, so Rps20 would have to dissociate, which is now limiting Rps29 binding. This of course is not Rps29-concentration-dependent. In addition, the finding that Rps20_EYER, which disrupts the Rps29/Rps20 interface (Collins et al., 2017), but has the opposite effect, is similarly consistent with the model. We have now changed the language in the manuscript to hopefully clarify this point.*

*“*Next, we used genetic analyses to obtain additional data to support a model that in wild type cells Rps29 binds before Rps20, and that in the Ltv1_L216S mutant Rps29 is partially excluded from 40S subunits because of a change in the order of binding. We have previously described a mutant in Rps20, Rps20_EYER, in which four amino acids at the interface with Rps29 are mutated (E80K, Y82A, E83K and R95E, [21]). If Rps29 binds before Rps20, then this mutant should impair the recruitment of Rps20; in contrast, if Rps20 binds before Rps29, then this mutant should impair the recruitment of Rps29 (**Figure S5E)**. We therefore devised a genetic strategy to distinguish between the two as described below.

We measured the effect from depletion of Rps29 on growth in wild type cells and yeast expressing Ltv1_L216S using yeast strains, where the Rps29 concentration can be reduced via addition of doxycycline (dox), and which express either wt Ltv1 or Ltv1_L216S. While reducing Rps29 concentration reduces growth in yeast with wt Ltv1, it has no effect on the growth of yeast expressing Ltv1_L216S (**Figure 6H**). This surprising finding strongly suggests that in yeast expressing Ltv1_L216S Rps29 association is not rate-limited by Rps29 association, but rather a step that is independent of Rps29 concentration, such as a folding step, or the dissociation of Rps20.

Next, we used the observation that depletion of Rps29 is epistatic with Ltv1_L216S, while depletion of Rps20 is synthetically sick with Ltv1_L216S, to test if the Rps20_EYER mutation behaves akin to the Rps20 depletion or the Rps29 depletion. Indeed, when we combine Rps20_EYER with Ltv1_L216S, we observe synthetically sick interactions (**Figure 6I**), akin to the observation with Rps20 depletion (**Figure 6G)**, and different to the effect from Rps29 depletion (**Figure 6H**). The observation that yeast with Rps29_EYER, which disrupts the Rps20/Rps29 interface, behave like Rps20 is depleted and not like Rps29 is depleted, suggests strongly that Rps29 is already present before this mutant binds, thus leaving Rps29 binding unaffected, while weakening the binding of Rps20 to the already prebound Rps29.

Strongly supporting these genetic interactions, are direct measurements of Rps20 occupancy in ribosomes from wt yeast or yeast expressing Rps20_EYER [26], which shows reduced levels of Rps20 in ribosomes from yeast expressing Rps20_EYER [26]. Moreoever, we have also shown that Rps20_EYER impairs the phosphorylation of Ltv1, akin to mutations that misposition Rps20 [21]. Thus, the reduced binding of Rps20 to ribosomes in the Rps29_EYER mutant is not consistent with Rps20 binding prior to Rps29, and instead supports binding of Rps29 prior to Rps20, as predicted from structural considerations (**Figure S5)**. In addition, the Rps29 concentration independence in the Ltv1_L216S mutant suggests that in this mutant Rps29 binding requires a step that is Rps29-independent, such as dissociation of Rps20, or remodeling of rRNA structure. Thus, together these data strongly support a model whereby in wild type cells Rps29 binds before Rps20, and where this order is perturbed in the Ltv1_L216S mutant, leading to the requirement for a conformational step (rRNA remodeling or Rps20 dissociation), and reduced levels of Rps29 in ribosomes. We note that future experimentation might reveal additional roles for Ltv1 in the recruitment of Rps29, including some that take advantage of the predicted direct binding between Ltv1 and Rps29 (**Figure 3)**.”

8. Similarly, if Ltv1_L216S masks the Rps29 binding site by over-stabilizing Rps20-Rps3 interactions, then mutations that weaken the Rps20-Rps3 interface (Fig. 6D,E), or low RP levels (Fig. 6F,G), should rescue this effect rather than exacerbate it, which is not what the authors observed.

*The Rps3_KK and Rps20_DE mutations that disrupt the interface between these two proteins have additional functional defects, which likely are the source of the synthetic negative interactions. First, like Ltv1_L216S they both impair Ltv1 phosphorylation (Huang et al., 2020). In addition, like Ltv1_L216S they also impair the folding of j34-35-38. Because this is likely due to the direct binding of these RPs to j34-35-38, this effect is predicted to be synergistic to the defect we observe here.*

9. I agree that the results show that mispositioning of mutant Ltv1 results in assembly defects in the head, including impaired recruitment of Rps29, but the evidence for the sequence of events cartooned in Fig. 8 is weak, in my view. It would be helpful if the authors can better explain the logic (report actual doubling times?) or reconsider their model.

*We believe the reviewer is actually referring to Figure 9B and have tailored our answer to this assumption.*

*Given that the entire order except the binding of Rps29 prior to Rps3 and Rps20 is based on our analysis of the cryo-EM structures whose PDB IDs are under the Figure, we assume that the reviewer is speaking directly about this particular part, which then would refer to the actual doubling times in Figure 6. As requested, this Figure now shows the actual doubling times.*

*Our conclusion that Rps29 binds prior to Rps3 and Rps20 is based on the following rationale. A) Rps29 depletion produces no growth defect in the background of the Ltv1_L216S mutation (Figure 6I); indicating that in this mutant Rps29 binding is limited not by association of Rps29, but a step that does not include Rps29 binding, such as a conformational step. B) The opposite is true for Rps3 and Rps20, and indeed this is the most typically observed effect, RP mutations are often synthetically sick with other RP of AF mutations. However, Rps20_EYER, which disrupts the interface between Rps20 and Rps29 has the same effect as depletion of Rps20, and not the same effect as depletion of Rps29; This suggests that it does the same as Rps20 depletion. Rps20 can only feel the effect from mutating the interface with Rps29 if Rps29 is already bound. Finally, these experimental data are also consistent with the structural analysis in Figure S5 (previously S4). We appreciate our lack of clarity and the poor choice in views in these Figures and thank the reviewer for their terrific questions, which have made this lack of clarity obvious to us. We have revised this section as described in the answer to question 8.*

10. One alternative is that Ltv1 increases the rate of productive Rps29 recruitment and thus the likelihood that it will happen before Rps20 binds. This would not require Ltv1 to act directly on Rps20 and Rps3. Another model is that Ltv1 embraces Rps29, preventing its dissociation but also requiring Ltv1 to move to permit Rps20 and Rps3 binding. If the mutant is less able to respond to Rps20 or Rps3, it would explain why the Ltv1 mutant is less sensitive to low levels of Rps29 (less dissociation of this protein) but more sensitive to Rps20 and Rp3 mutations.

*The first model suggested by the reviewer (increased rate of Rps29 binding) still predicts a concentration-dependence for Rps29, as it is a bi-molecular event involving Rps29. Thus, while this is an intriguing model, we feel it is not supported by the data. (However, it is also not ruled out by the data)*

*Similarly, the second model also predicts a bi-molecular step, and thus Rps29 dependence.*

*More broadly, it is plausible that Ltv1 plays additional roles in recruitment of Rps29 (or other RPs), but there are currently no data to support such additional roles, and the suggested model appears to be the simplest model that accounts for the data and is consistent with the previous finding (Collins et al.) that the contact between Rps3 and Rps20 is not formed when Ltv1 is present, and that Rps20 levels are reduced in the Rps20_EYER mutant. We have now clarified that additional roles for Ltv1 might be possible but that future experimentation would be required to uncover these.*

8. Please briefly indicate the methods used in the abstract (what type of data?).

*As requested, we have now updated the abstract to include this information.*

9. I appreciate that the authors mention the systematic ribosomal protein names in the text. It would be even better if they would use these names throughout. The reason is that this will make their work more accessible to readers working on human or bacterial ribosome biogenesis. The other reason is that prefixes “u” or “e” in Figure 1 or Figure 8 will instantly convey that the key ribosomal proteins in this study are universally conserved and define the (ancient) architecture of the 40S head.

*We have given this point considerable thought, both prior to submission of the manuscript, as well as upon reading the reviewer’s comment. In the end we feel it is better to use the eukaryotic nomenclature, precisely because this is a work that describes a human-disease-associated mutation, and the nomenclature in humans is nearly identical to the nomenclature in yeast, but both differ substantially from the bacterial nomenclature captured in the uS/eS nomenclature. Nonetheless, we entirely agree with the reviewer that the differing modes and roles that Ltv1 plays in the binding of eukaryote-specific and universally conserved RPs is nicely emphasized by the uS nomenclature. Thus, we have added the uS names throughout the introduction and within the discussion, and emphasized specifically how by binding universally conserved RPs Ltv1 modulated the assembly pathway to enable eukaryote-specific proteins to bind. In addition, we have highlighted the universally-conserved RPs in Figure 9B. We thank the reviewer for this comment, which made us think harder about Ltv1’s differential effects on eukaryote-specific and universally-conserved RPs.*

10. Pg 3, Rps5 is said to correspond to uS2; it should be uS7.

*We thank the reviewer for pointing out this mistake, which has now been corrected.*

11. Fig. 3 legend, “Ltv1 separates Rps20_DE from Rps3_KK” – do the authors mean that Ltv1 lies between these two proteins?

That is exactly what we mean. We have clarified this in the manuscript.

12. Fig. 3 legend, “Ltv1 binds Rps3K75R76” – does this refer to association of Ltv1 and Rps3 proteins, or does it refer to a predicted molecular interaction between Ltv1 and these particular residues of Rps3? The latter is not usually referred to as binding, which typically implies a bimolecular association.

*We thank the reviewer for pointing out the lack of clarity and how now updated the manuscript as suggested.*

13. Fig. 7 & 8 – consider colors for Tsr1 early and late with higher contrast.

*We have made the suggested change.*

**Additional review comments:**

To satisfy my own curiosity, I docked the alphafold predicted structure of yeast Ltv1 on the pre-40S cryo-EM structure (6FAI) as the authors did. After viewing the model, I realized that the interpretation of the mutational studies in this paper is much less justified than I initially believed. I now think that some of the assumptions in the paper are incorrect:

First, Ltv1 is predicted to adopt an extended structure that threads around and through the 18S 3′ domain (head).  This means that the alphafold prediction of the free Ltv1a protein, while useful, should not be taken literally, as it is likely that Ltv1 changes conformation as it binds the pre-40S complex.  Given the extended structure of Ltv1, I no longer think that the effects of the ribosomal protein mutations can be interpreted as revealing specific interfaces, only that the RPs and Ltv1 interact directly or indirectly in this region of the pre-40S complex (which we already knew).

*We could not agree more with the statement by the reviewer that the structure should not be viewed as an experimental result. We consider it a starting point and went to validate some of the predicted interactions. We disagree however with the sentiment that (i) we already knew about these predicted interactions and that (ii) the data in here do not provide information about direct interactions. While interactions between Ltv1 and Rps3, Rps20 and Rps15 were previously known, the interactions with Rps12, and j34-35-38 or l31 were not. Similarly, I would suggest that the location of the phosphosite at the side of the beak was unexpected, and so was the finding that Ltv1 is located under Tsr1 (although that explains a lot of biological data). In regards to (i), indeed, the observation that changes in the DMS accessibility overlap with sites of contact between Ltv1 and rRNA strongly suggest direct interactions. Moreover, we have also now analyzed changes in SHAPE reactivity from Hector et al., which essentially occur in vivo upon Ltv1 dissociation. Several of these also include residues directly contacted by Ltv1, as well as surrounding j34-35-38. These are now included as Figure S7. Thus, these further indicate that while obviously not the entire model is correct, broad strokes of the model are correct. Nonetheless, we agree with the general sentiment of the reviewer’s comment and have modified the language in the manuscript accordingly.*

Second, in the model I quickly generated, Ltv1 is predicted to clash with 18S h34, parts of uS3 (rps3), an extended loop of uS10 (rps20), and much of uS14 (rps29). This contradicts the presumption that Ltv1 “separates” uS10 from uS3 while they are bound to the pre-40S; these three proteins cannot co-exist in the structure as modeled. Thus, while Ltv1 likely plays a direct role in chaperoning the complex RNA and protein interactions in this region of the 40S ribosome, it is also highly likely that Ltv1 must change structure as the RPs bind the 18S rRNA.  The details of such rearrangements cannot be resolved by genetics experiments, or the DMS footprinting data in this paper.

*We thank the reviewer for providing this strong analysis, and entirely agree. In fact, we are working on a follow-up paper that starts to address some of these molecular changes. Previous biochemical data indicate that Rps3 and Rps20 are not making their contact, as described in detail, yet the available structures show a later intermediate that has these already interacting. Indeed, our previous data in Huang et al., 2020 demonstrate changes upon formation of the Rps20, Rps3 contact, as indicated by the reviewer’s comment. We have made this more clear in the manuscript as described below. “*The observation that Ltv1 blocks the formation of the contact between Rps3 and Rps20 also suggests that Ltv1 must move to enable the formation of this contact and allow for its phosphorylation by Hrr25 [21], indicating that flexibility in Ltv1 might be functionally important.”

Third, on pg. 9, as justification for the experiments in Fig. 6, the authors state that uS14 lies under uS10 and therefore uS14 must bind first. In the cryo-EM structures of the mature yeast ribosome or the pre-40S ribosome, uS14 lies OVER an extension of uS10. The parsimonious binding order is uS10 > uS14 > uS3. The interpretation of these results should be revisited and the text substantially revised.

*The images to illustrate this were poorly conceived, and we have now revised them. Importantly, when one looks from the backside, there is a small piece of Rps29 that is in front of Rps20. However, when one looks from the top, which is where the opening is, and where one must assume that Rps29 and Rps20 enter from, then it becomes very clear that Rps29 is located under Rps20. This is now shown in new panels in Figure S5C and D. In addition, the genetic data that demonstrate that weakening the Rps20/Rps29 interaction (Rps20_EYER) has the same genetic interactions as depletion of Rps20, but the opposite interactions as depletion of Rps29 indicates that Rps29_EYER affects Rps20 binding. This can only be the case when Rps29 is already there. Thus, together the genetic and structural data, together with published biochemical data, strongly support the proposed model.*
